# Crossing survival curves: alternatives to the log-rank test

**DOI:** 10.1186/1745-6215-12-S1-A137

**Published:** 2011-12-13

**Authors:** George Bouliotis, Lucinda Billingham

**Affiliations:** 1 MRC Midland Hub for Trials Methodology Research, University of Birmingham, Birmingham, B15 2TT, UK; 2 Cancer Research UK Clinical Trials Unit, University of Birmingham, Birmingham, B15 2TT, UK

## Background

It is not uncommon for clinical trials to present results on survival time as Kaplan-Meier survival curves that cross, indicating non-proportional hazards. A recent example was given in a pivotal trial in advanced non-small cell lung cancer (The ‘IPASS study’ [1]). Trials such as these present a hazard ratio and log-rank test for treatment comparison as this is their planned primary analysis. However, the validity of such analysis is questionable and has received published criticism. This paper reviews the use of the log-rank test with crossing curves and considers alternatives that have been proposed.

## Methods

The review of the alternative approaches includes weighted log-rank tests (Wilcoxon, Tarone-Ware, Peto-Prentice and Fleming-Harrington), supremum versions of the log-rank test (modified Kolmogorov-Smirnov and Renyi-type tests) which are based on the maximum difference between estimates of two survivor functions and modified log-rank tests (Lin and Wang test using squared differences at each time point, and Levene-type test focusing on variance differences). In addition, methods based on splitting the analysis at the crossing point have also been proposed. Methods are compared and evaluated using both real and simulated datasets using Weibull and Weibull-Cox distributions representing realistic situations.

## Results

Crossing survival curves is generally a result of the survival times having greater variance in one treatment group than another. The performance of the log-rank test and alternatives depend on the type of crossing (early, mid or late) but in general the probability of a Type II error is increased for log-rank and weighted log-rank tests but performance is improved with the alternatives. The choice of time-point for the split-analysis is problematic. Standard software such as sts test (Stata), proc lifetest (SAS) and survfit (R) and routines-on-demand support some but not all the tests considered.

## Conclusions

There is a need in the clinical community to clarify methods that are appropriate when survival curves cross. Statistical analysis plans for clinical trials with survival as primary outcome measure should specify an analysis dependent on the proportionality of hazard rates and explicitly consider non-proportionality issues, powering the analyses based on log-rank alternatives. Modelling the survival data may be more appropriate than simple univariate hypothesis tests when hazards are not proportional. Finally, there are some feasibility issues regarding software for such analysis that remain to be tackled.

## References

[B1] Mok TS Wu YL Gefitinib or carboplatin-paclitaxel in pulmonary adenocarcinoma New England Journal of Medicine 2009 361 10 947 57 10.1056/NEJMoa0810699 19692680

